# Otocephaly: A Case Report of a Rare Congenital Anomaly

**DOI:** 10.7759/cureus.41767

**Published:** 2023-07-12

**Authors:** Hameed Ur Rahman, Aleena Anees, Muhammad Asfandiyar Ali, Saad Ahmad, Abu Baker Khan

**Affiliations:** 1 Internal Medicine, Ayub Teaching Hospital, Abbottabad, PAK; 2 Pediatrics, Ayub Teaching Hospital, Abbottabad, PAK; 3 Orthopedic Surgery, Taj Medical Center, Nowshera, PAK

**Keywords:** antenatal scan, congenital disorder, microstomia, agnathia, otocephaly

## Abstract

Otocephaly is a rare congenital abnormality characterized by the absence or underdevelopment of the mandible, misplacement of the ears towards the front, a small mouth, and absence or underdevelopment of the tongue. The syndrome complex of otocephaly can be categorized into four types based on associated anomalies. We present a case of this congenital anomaly in a newborn baby delivered by a 40-year-old woman who presented in active labor with premature rupture of membranes. Unfortunately, the newborn did not survive due to severe respiratory distress, which was consistent with the clinical features of this congenital anomaly. The rarity of otocephaly poses challenges for both parents and healthcare providers. Early antenatal scans are suggested for the early diagnosis of this condition. Further research and awareness are needed to better understand and manage this rare congenital disorder.

## Introduction

Otocephaly is an uncommon abnormality characterized by the absence or underdevelopment of the mandible (agnathia), misplacement of the ears towards the front (melotia), a small mouth (microstomia), and the absence or underdevelopment of the tongue (aglossia or microglossia) [1]. This infrequent anomaly affects the frontal part of the first brachial arch and occurs due to improper migration of neural crest cells from the hindbrain [1,2]. Its origin has been linked to both genetic and teratogenic causes. Factors such as salicylates, theophylline, radiation, and alcohol exposure during pregnancy have been reported as potential causes [2]. Respiratory problems are common in affected individuals, and the spectrum of malformation is often lethal. The most commonly associated condition is holoprosencephaly, although skeletal, genitourinary, cardiovascular anomalies and situs inversus have also been reported [3]. Given the rarity of this congenital anomaly, the significance of our case report lies in highlighting this clinical entity.

## Case presentation

**Figure 1:**
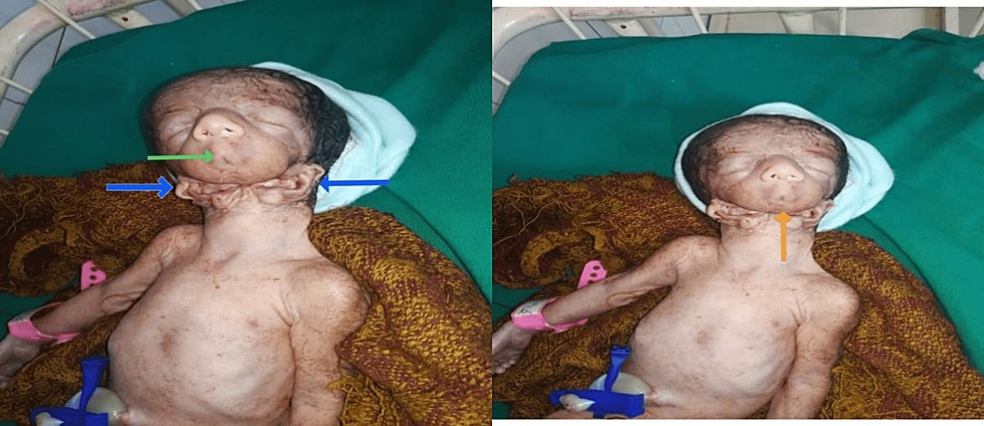
Microstomia (green arrow), ventromedial position of ears (blue arrows), and mandibular hypoplasia (orange arrow).

A male baby, born at a government hospital via normal vaginal delivery, presented at 35 weeks gestation with a weight of 2000 grams. The mother, aged 40 and multigravida, had previously delivered four healthy children without complications, all through normal vaginal deliveries handled by midwives. She was admitted to the hospital due to complaints of premature rupture of membranes. During labor, the mother delivered the baby without medical assistance. The family resided in a rural area, and the mother neither sought obstetric consultation during her pregnancy nor used any supplements. Antenatal ultrasounds were not available upon her arrival at the hospital. The newborn baby exhibited severe respiratory distress upon birth and was immediately transferred to the neonatal intensive care unit (NICU). The baby was bradycardic and showed no signs of breathing efforts. The newborn was administered bag and mask ventilation, but resuscitation efforts were unsuccessful. Upon examination, the baby displayed distinctive facial features. He had a small mouth (microstomia) with an absent mandibular bone (agnathia), resulting in an elongated appearance of the neck. The bilateral ears were positioned ventromedially on the neck and appeared fully developed, with proper helix and lobule. However, the lobules and concha of the ears were fused with the skin of the neck region at the ventromedial position. The baby's upper limb and thorax were normally formed (Figure 1). 

To rule out any tracheoesophageal anomalies, a nasogastric tube was passed through the nose and mouth. The tube was confirmed to be properly placed in the stomach through auscultation, and no associated abnormalities were noted. The mother denied any intake of teratogenic medications during pregnancy, and there was no positive family history of this congenital anomaly. The parents were informed about the possibility of an autopsy but declined due to social and ethical reasons.

## Discussion

Otocephaly is an exceedingly rare condition characterized by facial abnormalities, including mandibular hypoplasia or agnathia, ear anomalies (melotia, synotia), microstomia, and aglossia [1]. Another related condition known as agnathia otocephaly complex is diagnosed when an underdeveloped or absent mandible, misplaced ears with or without auricular fusion, and a small mouth with oroglossal hypoplasia or aglossia are present. The prognosis can be significantly influenced by additional features such as holoprosencephaly, cyclopia, anencephaly, meningomyelocele, situs inversus, lung and genitourinary anomalies, and skeletal and cardiovascular abnormalities [2]. The exact cause of agnathia otocephaly complex remains unclear but is believed to be associated with genetic and environmental factors [4]. Certain genes, including OTX2, PRRX1, and CRKL, have been identified as potential contributors to agnathia. The expression of the transcription factor OTX2 in mesenchymal cells of the midbrain, head, and neural crest cells has been shown to cause craniofacial malformations and holoprosencephaly [5]. In addition to genetic factors, the use of drugs such as theophylline and salicylic acid, among others, has been implicated in increasing the risk of this condition [5]. However, in our case, the mother did not take any such drugs during pregnancy, and no prenatal ultrasound was performed. Prenatal ultrasound serves as an effective screening tool for diagnosing agnathia otocephaly complex [3]. However, accurately identifying mandibular development and identifying auricles on prenatal ultrasound can be challenging. As a result, prenatal diagnosis of the disease remains difficult [3,6,7]. The mandible can be specifically assessed using sagittal and transverse sections of the face. Additionally, the mother did not take any supplements during pregnancy. Bakhit AM et al. report a similar case of otocephaly born to a 40-year-old lady in Egypt. Both underscore the severe consequences associated with otocephaly, a rare congenital anomaly. Despite marked differences in the mothers' reproductive histories and the availability of prenatal care, both babies manifested common features of the condition, including microstomia and agnathia. In terms of facial anomalies, our case had an interesting presentation of synotia with fully formed ears fused to the neck skin. Despite attempts at resuscitation, both neonates, unfortunately, succumbed soon after birth [8]. This comparison illustrates the varied etiology and presentations of otocephaly, underlining the importance of comprehensive and accessible prenatal care, particularly the use of antenatal ultrasound in early detection. It further calls attention to potential associations between otocephaly and advanced maternal age or assisted reproduction techniques. Similar cases have also been reported in the literature [5-9]. Long-term survival is nearly impossible due to the poor prognosis associated with the agnathia otocephaly complex [9]. Infants born alive typically succumb to respiratory distress resulting from the absence of a supportive mandible, which leads to the underdevelopment of crucial adjacent structures, particularly the naso-mandibular complex, and oropharynx. This respiratory distress, coupled with feeding difficulties, speech, and hearing impairments, further compounds the challenges faced by affected individuals [10]. Even in cases where survival is achieved, assisted breathing via tracheostomy and assisted feeding through a gastrostomy tube remain necessary [11]. In our case, the infant passed away due to severe respiratory distress. To ensure the mother's and fetus's safety, prenatal 2D and 3D ultrasound examinations are essential for identifying facial defects, followed by genetic studies to rule out such conditions.

## Conclusions

Otocephaly presents a significant challenge for parents and healthcare providers due to its rarity and complexity. Early antenatal scans play a crucial role in identifying this congenital anomaly. In addition to the advancements in normal facial screening, we suggest incorporating mandibular arch screening during the first and early second-trimester evaluations. Early diagnosis of this lethal abnormality is valuable as it allows for safer termination of pregnancy when desired. Timely reporting of suspected cases is essential to raise awareness among healthcare professionals and provide sufficient time to conduct genetic studies and research, leading to a better understanding of this rare congenital disorder.
